# Letter from China

**Published:** 1874-05

**Authors:** 

**Affiliations:** Shanghai China


### Correspondence.

SHANGHAI CHINA, Feb. 12th, 1874.

TO THE EDITOR OF THE DENTAL REGISTER:

*Dear Sir*:—Having received many letters from dentists in the United States, making inquiries concerning the advisability of coming to China and Japan to establish a Dental Practive, and being unable to answer all such in detail, will you kindly allow me a short space in your valuable Journal to reply to all whom it may concern. Let me premise, however, by saying that, one is often at a loss to know how there should be so lamentable a lack of knowledge in some persons, of the real status of things in the East, the more especially, as a regular line of steamers carrying the United States mails from China and Japan to the United States has been in existence for over five years—but as such want of knowledge really exists, it will be the object of this paper to put things in their real light, so that he who runs may read and learn.

In the first place, then, the field is more limited than most suppose, and to give a perfectly correct account, I shall quote from a directory of China and Japan before me, including the principal ports open to foreign intercourse. First, I shall mention Hong Kong—a British colony; with a governor and his legislature. It is a place of about one hundred thousand inhabitants, including all nationalities. But of these, the Chinese number about eighty thousand, the Portugese some eighteen hundred, and the foreigners proper about eleven hundred and i. e, Englishmen about eight hundred and fifty, Germans abuot one hundred and fifty and Americans about one hundred. Now, a dentist has to rely soley for his practice upon these last three classes—the Chinese never seek a foreign dentist's services, except in very rare instances among the wealthier classes ; and during the writer's residence of nearly six years in Hong Kong he never did more than $300 worth of operations for the Chinese. As to the Portugese, they are a very inferior class of people with about eight tenths of Chinese blood, and are a class utterly without means or influence in any way, and occupying the most menial positions and occupations. So that in fact, a dentist has to rely entirely upon those foreign elements before mentioned. This fact therefore shows that a dental practice is to be made and sustained among about eleven hundred persons in Hong Kong. Secondly, I shall speak of Shanghai, as being the largest open port in China. The population of Shanghai is about two thousand, three hundred foreigners, classified exactly as was that of Hong Kong, with the exception that the strictly foreign element is abont five hundred souls greater. Then comes the "coast ports" including "Swa-tow," "Amoy" and "Foo-chow." Of these, the latter is much the largest numbering about three hundred and fifty Foreigners, all told, and equalling in numbers both Amoy and Swa-tow. Having spoken of the population of the several principal ports in China, I now speak of the occupants of the same. In Hong Kong there are now two regularly established American dentists, both men who have come out under special agreement to succeed long established dentists (the one of whom has left China on account of ill-health—the other moved to a new field)—as to Shanghai—there are now two dentists here, one long established, the other trying to build up a practice. Now as the Hong Kong dentists divide their time between the latter place and the "coast ports," it is clearly evident that the entire field in China is more than fully occupied. The same is true of Japan as of China. Yokohama and Kobe are the only ports of the least importance in Japan, with a foreign population of about seven hundred, all told, and this field too has long been occupied by Dr. Elliott, an American dentist of high professional and social standing.

From the above statements, it can be clearly seen that these two fields, China and Japan, have no room at all for any new comers. Now, as to the practice in these two fields. It is emphatically a peculiar and trying one. The English predominate in the East generally in the proportion of two thirds the entire foreign element, and to establish a practice among them requires a peculiar fitness in many ways. To succeed, a dentist must be able to take a high professional and social standing, and be able to stand side by side with thoroughly educated medical men, with very high notions of professional status and etiqutte. Then again, the English are very clanish, and run entirely in grooves, but when they get the confidence of a certain dentist, he and he only gets their entire patronage. Again, the cost of living is excessive here, so that no dentist may expect to keep house and a respectable office for less than from $5000 to $6000, per annum, and in fact, can live in any of the large cities at home for less than it costs here. These last two facts, the difficulty of getting the public to go to a new man, and the cost of living, have been the rock on which many dentists have been wrecked in the East during the nearly 9 years' residence of the writer. Within the last five years, no less than six American dentists have come and gone, and in the case of four of these public subscriptions have been raised in order to send them back home, poorer (if not wiser,) men than when they left respectable practices at home to "try their fortunes" in this part of the world. The climate too, is trying and wearing, and few stay longer in China especially, than ten years. Then to a married man, there is to be added to the fact of a limited field the difficulties of good schools for his children, and it generally amounts to this, that after a residence of a few years, wives go home with children to be educated and recuperated in health, and husbands stay to plod on, separated from family, and put to the expense generally of keeping up two homes—the one for wife and children at "home," and the other in China. The expense also of ge ting out is an itcm, as a man coming from Cincinnati say, can not reach China short of from $900 to $1000. In conclusion therefore, let me say to all who are premeditating coming to the East, to better themselves in this worlds goods, stay at home. There is no better field in the wide world for a professional man, whether he be dentist or some other profession, than the broad and great United States. To be "forwarned, is to be forearmed," therefore let a word to the wise be sufficient.

Yours truly
Dentist.

